# Evaluation of real-time monitored ozone concentration from Abuja, Nigeria

**DOI:** 10.1186/s12889-023-15327-1

**Published:** 2023-03-15

**Authors:** Christabel Ihedike, John D. Mooney, John Fulton, Jonathan Ling

**Affiliations:** 1 Faculty of Health Sciences & Wellbeing, University of Sunderland, Sunderland, England; 2 University of Aberdeen, Aberdeen, Scotland

**Keywords:** Real-time ozone monitoring, Air pollution, Nigeria, Abuja, Harmattan

## Abstract

Real-time ozone (O_3_) concentration is vital for accurate analysis of O_3_ to inform the public about O_3_ concentrations that may have an adverse effect on health. Few studies have analysed air pollution in Abuja, Nigeria and non on real-time ozone concentrations. As a result, there is a scarcity of data and information on real-time ozone pollution, pointing to a gap that needs to be urgently closed to enable a better understanding of ozone pollution and the causes and consequences in terms of the associated health risks.

In this study, -time concentrations of ground-level ozone were measured in a busy urban pollution monitoring station. Using a real-time ozone monitor to enable real-time monitoring of O_3_ concentration of ozone for the first time in Abuja. The ozone concentrations followed a clear pattern with high concentrations being recorded during the dry (harmattan) season. Concentrations higher than the WHO standard of (eight-hour averaged) 100 µg/m^3^, occurred on 53 days over the 5-month dry season. Of those 53 days, 18 had ozone concentrations greater than 200 µg/m^3^. Daily patterns showed a rise throughout the day, reaching a peak in the evening. Weekday/weekend differences were less pronounced than those found in other studies. High temperatures and local climatic conditions in Abuja encourage the formation of ozone. In this study, we confirm the concentration of ozone, and the pattern can be episodic and potentially damaging to health. There is a need for better regulation and measures to reduce ozone, particularly when local climatic conditions, such as harmattan, favour the development of photochemical smog in such settings.

## Introduction

Good air quality is one of the basic needs of human existence. However, air pollution continues to cause substantial threats to health. The World Health Organization [1] has calculated that over 2 million premature deaths yearly are attributed to air pollution and these effects are more predominant in developing countries. Ozone (O_3_) is one of the most potentially damaging air pollutants. Ozone is a major constituent of smog that results from photochemical reactions of oxides of nitrogen and volatile organic compounds [2, 3]. High ground-level ozone concentrations are problematic because of their potential health effects. Ozone presents a difficult control problem because it is a gas created in the atmosphere and not directly emitted from processes that can be regulated, and its creation can take place over a wide range of time and distance [4, 5].

Both indoor and outdoor air quality are closely related to morbidity and mortality from respiratory and cardiovascular diseases. [1] Epidemiological studies indicate that the rates of asthma attacks and medication usage increase on days with higher O_3_ concentrations. The rates of hospital emergency room visits and hospitalisation for asthma and other respiratory conditions are also increased on such days [6, 7]. While many studies have been conducted in the developed world [8, 9] exposure assessment has been under-evaluated in the developing world [9]. This is primarily due to a lack of air pollution monitoring data, and consequently, health impact studies cannot be carried out. Without these studies, the development of land-use and transportation policies to improve air quality is challenging [10].

Ozone pollution is a major challenge in developing countries. The causes of ozone pollution in developing countries are diverse, but one of the main reasons is economic growth [11] with expanding industrialisation and increasing traffic volumes leading to an increase in emissions of many air pollutants including those that are precursors for ozone formation [11, 12]. While the relationship between ozone formation and increasing temperatures is complicated, warmer temperatures are likely to increase O_3_ concentrations [13]. Although future O_3_ concentrations can be modeled and projected, air quality monitoring data is needed to provide baseline information to reliably estimate future possible concentrations. Such data are, however, missing for many Sub-Saharan African countries.

Nigeria has both abundant fossil fuel resources and a poorly maintained energy infrastructure leading to a reliance on home generators, this situation has resulted in elevated localised emissions of both NO_x_ and VOCs [14]. The WHO [15] guideline for O_3_ is a daily maximum 8-hour mean of 100 µg m^− 3^. The WHO noted that above 240 µg m^− 3,^ there would be significant health effects in a substantial proportion of the vulnerable population.

Our aim was to obtain real-time data to make comparisons with health-based standards and provide health information to vulnerable groups. Previous work in Nigeria has used chemical absorbents to collect O_3_ every month at ground level. For example, such methods used in Ibadan, Nigeria recorded a monthly mean of 31 µg m^− 3^ [16]. Such methods, however, may be inaccurate and also affected by the time of day that the data are collected. In the present study, we collected real-time O_3_ data using a monitor sited at the Nigerian Meteorological Agency (NIMET) monitoring station in Abuja, Nigeria. The data were collected as a part of an epidemiological research project to investigate the effects of O_3_ on people with respiratory health problems.

## Methods

### Study area

**Fig. 1:**
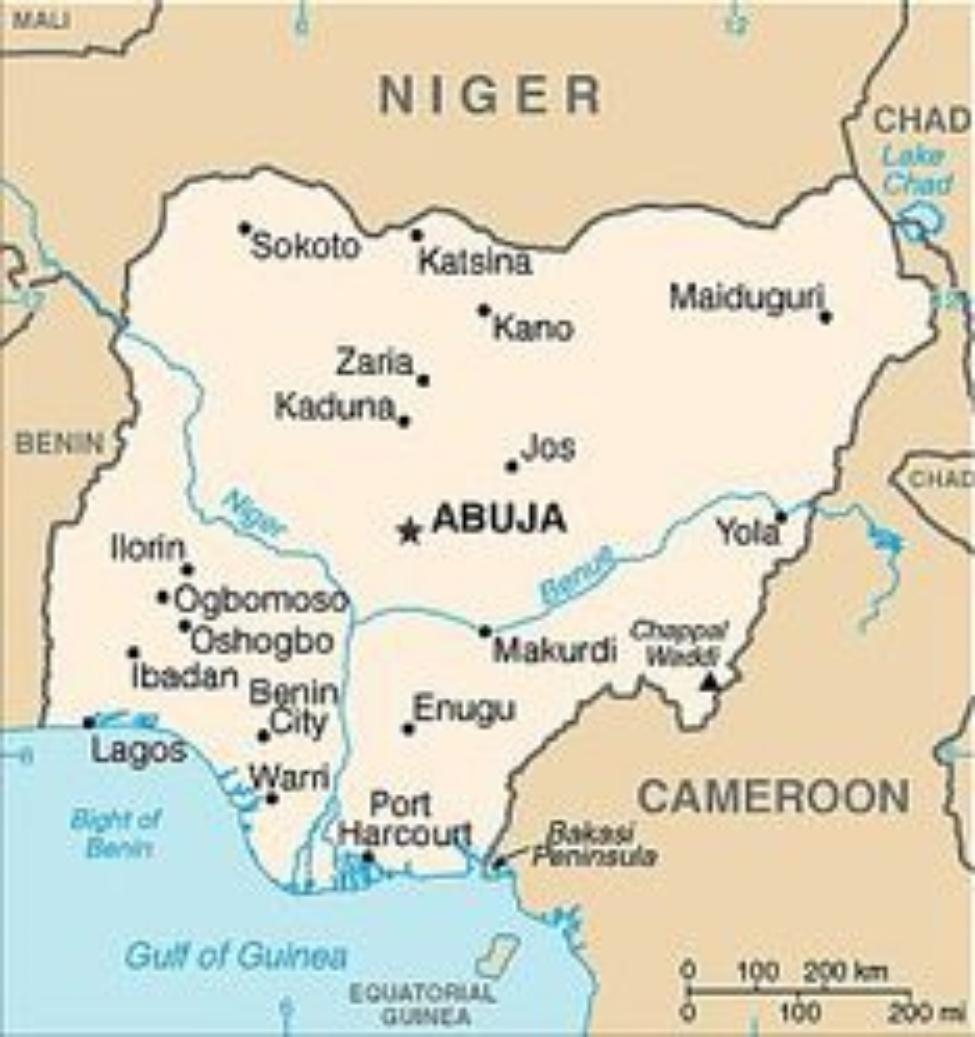
Map of Nigeria showing Abuja. (source World Atlas)

The research was conducted in Abuja, the federal capital territory, situated at the center of Nigeria (Fig. 1) covering 7,754 km^2^. Abuja’s population is about 3,277,740 people and covers a range of people from civil servants to farmers [17]. According to the World Population Review [17], Abuja is a fast-developing city with high road traffic. The need to evaluate real-time O_3_ concentration in Abuja is vital because of the growing population, use of electric generators, bush burning, mining, and emission of NO_X_ and hydrocarbons, among other factors. Also, exhausts from vehicles contribute notably to the concentration of pollutants in the air. Consequently, this causes a high rate of NO_x_ and PM_10_ concentrations, as well as the potential for the formation of O_3_ [18, 19].

Typically, Nigerian weather has two distinct seasons: a rainy season (May to October) and a dry season (November to April) [20]. The dry season features brief intervals, known locally as the Harmattan periods, when cold and dust-laden north-easterly winds, from the Sahara Desert, transport significant dust quantities for multiple days. This period is usually dry with high solar radiation and clear sky conditions, moderate air temperatures, and no precipitation [21].

The NIMET monitoring station is in an office complex in the center of Abuja. The surroundings of the site are mainly commercial buildings and roads. The station is 8 m away from a major road. The NIMET station monitors concentrations of PM_10_, CO, NO_x,_ and SO_2_. A monitor for O_3_ (2B Technologies Model 202 Ozone Monitor supplied by Air Monitors, UK) was supplied from the UK for this study. The monitor uses the absorption of UV light at 254 nm to measure the concentration of ambient ozone. The monitor has an extra battery for power and low power consumption. The ozone monitor was maintained and calibrated by NIMET according to the manufacturer’s instructions. However, during this study, NIMET overhauled its monitoring site and could not monitor other pollutants other than ozone.

### Data collection/air monitoring

The author with the support of NIMET staff used the Abuja NIMET monitoring station and measured the ozone concentrations throughout the study period. The ozone was recorded every 5 min and was calculated to 1 h and 8-hour running mean and monthly mean reflecting the WHO and Federal Ministry of Environment proscribed limits.

## Results

The ozone data obtained from the monitor were compared with ozone data from the other months of the year to determine the concentration and compared against World Health Organisation air quality standards.

**Fig. 2:**
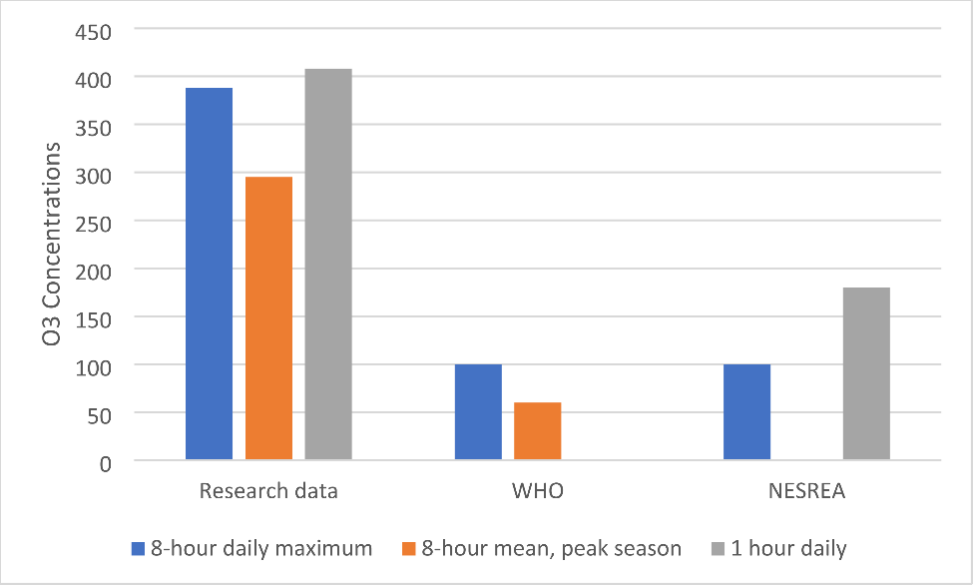
Monthly maximum, mean, minimum, median and mode in 2018

**Fig. 3:**
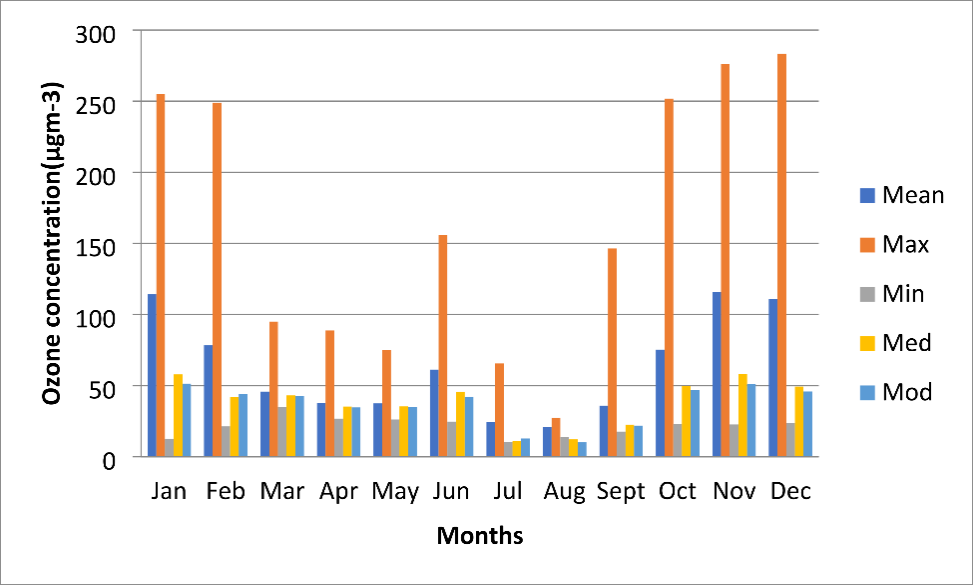
8-hourly max, mean, and hourly mean of O_3_

The ozone data show varying ozone concentrations for Abuja residents with concentrations above the limit on most days. Table 1 shows the monthly mean (+/- sd) ozone, and Fig. 2 Mean, maximum, minimum, median, and mode of ozone concentrations across the year. Table 2 shows some days in certain months when the daily maximum 8-hour mean O_3_ > 100 µg m^− 3^ and > 200µ g m^− 3^. The data were compared to both air quality standards and published data in Fig. 3.

The data during this study showed that the highest ozone concentration recorded in Abuja was 388 µg/m^3^ (1-hour mean) and 295 µg/m^3^ (8-hour mean). The ozone concentration during data collection was almost three times higher than the WHO-recommended standard for O_3_ (100 µg/m^3^ 8-hour mean) (WHO, 2005 and 2021b) for both the 1- and 8-hourly means.

Figure 2 shows different levels of ozone pollution in Abuja. This could be attributed to many sources including open burning, traffic, and meteorological conditions. As Abuja is the administrative capital of the Federal Capital Territory. It commonly experiences high traffic flow during the day, particularly during commuting rush hours (morning and evening).

**Table 1:** Monthly Mean (+/- SD) Ozone µg m^− 3^ measured at NIMET Abuja monitoring site between January 2018 and December 2018

Month	Monthly mean O_3_ concentration (µg m^− 3^)	+/- SD
January	114.3	57.5
February	78.4	48.3
March	45.8	1.7
April	37.8	3.0
May	37.5	3.2
June	61.2	26.9
July	24.3	15.3
August	20.9	8.8
September	35.9	21.3
October	75.3	46.8
November	115.7	58.6
December	111.0	55.8

The monthly mean ozone concentrations are most substantial during the dry season (October to February) when monthly means greater than 70 µg m-^3^ were recorded. During the wet season (March to September), monthly mean concentrations reduced to below 45 µg m-^3^. There was an exception for June when a monthly mean of 61.2 µg m^− 3^ was recorded. The data recorded during the wet season was similar to the monthly mean ozone concentrations of 31 µg m-^3^ recorded in Ibadan [16]; the concentrations in January, November, and December were far higher. However, the monthly ozone concentration mean levels are lower than in Fig. 3 because these are 24-hour readings, so they are reduced by lower traffic and temperatures.

January is among the dry season months. The maximum, mean, minimum, median and mode observed concentrations show that ozone was high in Abuja atmosphere during this season.

**Table 2:** Number of days in each month when the daily maximum 8 h mean O_3_ µg m^− 3^ was 100–200 and > 200

Month	Number of days with an 8-hour mean 100–200 µg m^− 3^	Number of days with an 8-hour mean > 200 µg m^− 3^	Number of day wih 8-hour mean100 + µg m^− 3^ (col1 + col2)
January	6	6	12
February	5	2	7
March	0	0	0
April	0	0	0
May	0	0	0
June	5	0	5
July	0	0	0
August	0	0	0
September	0	0	0
October	5	2	7
November	2	6	8
December	12	2	14
Total for 2018	35	18	53

One of the problems in determining monthly mean values for ozone is that this does not record episodes of high ozone concentrations. The difference in ozone data and episodes are more prevalent on days of higher ozone concentrations. The number of days on which an 8-hour mean greater than 100 µg m^− 3^ (the WHO standard) and also 200 µg m^− 3^ (a concentration likely to have significant effects on health), shows the same seasonal variability as seen in the monthly mean data (Table 2). Except for June, there were no exceedances (above WHO limits) during the wet season months of March – September. During the dry season, there were several days when ozone episodes peaked above 100 µg m^− 3^ with some exceeding even 200 µg m^− 3^. In 2018 there were 35 episodes during the dry season when concentrations were between 100 and 199 and 18 episodes when concentrations were greater than 200 µg m^− 3^. Ozone peak season as observed in this study is 283.3 µg m^− 3^ and is higher than the WHO new standard peak season of 60 µg m^− 3^ [22].

O_3_ concentrations, for both weekdays and weekends, start to rise at 7 am reaching a maximum between 2 pm and 8pm, and decrease until midnight. These results indicate that O_3_ maximal concentrations in Abuja occur slightly later in the day.

## Discussion

**Fig. 4:**
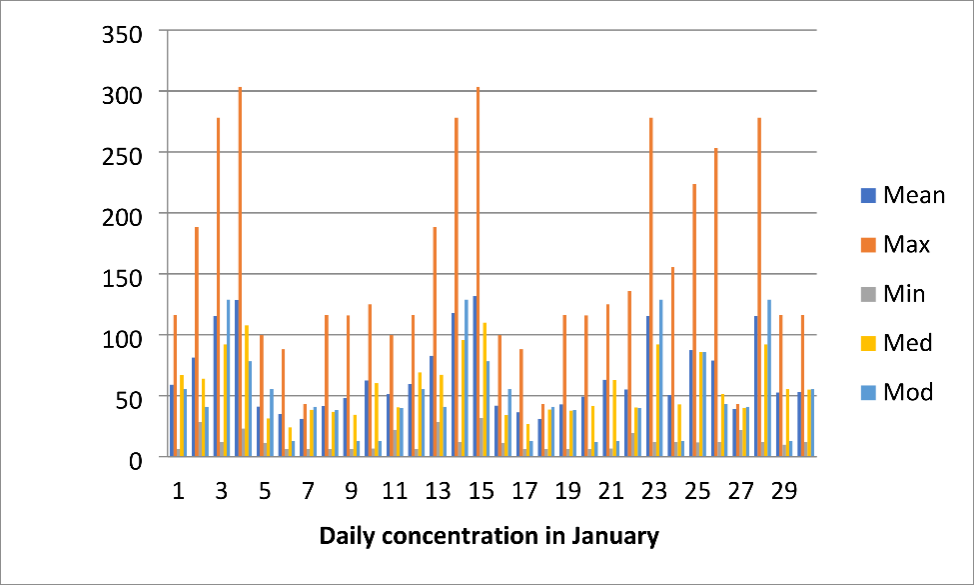
Hourly mean O_3_ µg m^− 3^ recorded on weekends and weekdays in December 2018

In Abuja, high ozone concentrations occur throughout the dry season and last for periods of 1–3 days. There is little difference in the hourly concentrations of O_3_ measured at weekends and weekdays (see Fig. 4). This differs from previous research where reductions in industrial and transportation activities led to lower concentrations of NO, PM, and O_3_ at weekends [12]. This suggests no difference in the sources of pollutants over the days of the week and that the dates on which exceedances occur are primarily driven by other factors such as the weather. Predicting these episodes would enable health warnings to be issued, which would be particularly important for individuals vulnerable to the effects of high levels of air pollution. The high concentrations/episodes have been associated with effects on humans, materials, and vegetation [23–25]. Although measurements of ground ozone are limited in Africa [26].

Ozone concentrations usually show a daily pattern of increases in the morning, reaching a peak in the late afternoon and decreasing through the evening to reach a minimum at night [27]. We observed a similar pattern in Abuja. Concentrations, for both days, start to rise at 7 am reaching a maximum between 2 pm and 8pm, there is then a decrease until midnight. This indicates that O_3_ maximal concentrations in Abuja occur slightly later in the day when compared to other cities, for instance, Guadalajara, Mexico, where maximum O_3_ concentrations were recorded between 13.00 and 16.00 [27].

**Fig. 5:**
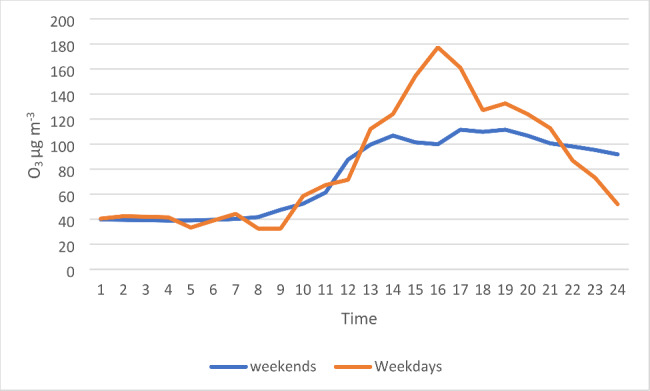
Daily maximum, mean, minimum, median and mode in January 2018

Also, studies on ozone measurements in southwestern Sub-Saharan Africa have shown that outdoor ozone concentrations regularly exceed WHO guidelines and increasing ozone concentrations are observed during summer [28]. A clear diurnal variation was observed in this study which is similar to some studies in southwestern Sub-Saharan Africa with continuous monitoring of O_3_ concentration increasing from a minimum near sunrise to a maximum in the afternoon, after which concentrations decrease again to the early morning minimum [26]. Also, the daily maximum, mean, minimum, median, and mode of O_3_ concentration in January in this study (Fig. 5) were higher than reported in other studies [12, 26].

In Sub-Saharan African countries there is a scarcity of studies when it comes to measurements of ground-level O_3_ concentrations [26]. However, the outcome of this study is similar to the few conducted in Sub-Saharan Africa for example, O_3_ concentrations exhibit strong seasonal and diurnal variations with the maximum occurring in the months from October to February and the minimum in March to September (Table 2). A strong diurnal variation also occurs with ozone concentrations increasing from a minimum near sunrise to a maximum in the afternoon, then decreasing again to the early morning minimum.

The health effects of ozone are reversible, with recovery and improvement to baseline fluctuating from a few hours to 48 h after increased O_3_ exposure. The newly revised WHO Air Quality Guidelines have established a guideline value for ambient air of 100 µg/m^− 3^ for a maximum period of 8 h per day as a level at which acute effects on public health are likely to be experienced [22]. However, people may experience some impact on their health even at ozone concentrations around or below this guideline value. The risk of these outcomes is estimated on the basis of the tables provided with the guidelines [15, 22]. For example, 8-hour exposure to ozone at a concentration of 100 µg/m^− 3^ is expected to induce 5% (transient) decrement in pulmonary function in an active healthy individual, and the most sensitive 10% of young adults and children; at 160 µg.m^− 3^ this decrease is expected to be more than 10%, which may lead to health problems even in healthy individuals. Furthermore, an increase in 8-hour average ozone concentration of 100 µg/m^− 3^ is expected to induce 25% increase in symptom aggravation among adults and asthmatics involved in normal activities and a 10% increase in hospital admissions for respiratory conditions [8, 9, 11].

These increases in the risk of health effects assume linear relationships between ozone concentration and health effects. However, a great deal of uncertainty exists with respect to the shape of these relationships. The health benefits of reduction of exposure to high concentrations of ozone are greater than the same absolute reduction of exposure in the lower concentration range. Although chronic exposure to ozone can cause effects, quantitative information from humans is inadequate to estimate the impact of long-term exposure [29, 30].

In Abuja, mean monthly ground-level ozone concentrations follow a seasonal pattern with the highest concentrations being found during the dry season. However, whilst the monthly concentrations are higher than during the wet season, daily ozone concentrations are not always high during the dry season. Episodes of very high concentrations, greater than 100 µg m^− 3^, occurred on 53 days during the 5-month dry season period. This is above the 3–4 exceedances recommended by the WHO per year [22]. Of those 53 days, there were 18 days when the highest 8-hour mean was above 200 µg m^− 3^ a concentration known to impact health [1, 15, 22, 31]. Also, O_3_ concentration during data collection was almost three times higher than the WHO recommended standard for O_3_ (100 µg/m-^3^ 8-hour mean) [15, 22] and the Nigerian standard for both the 1- and 8-hourly mean [32].

It is important to note that O_3_ formation is dependent on both sources of the precursors and atmospheric chemistry. The atmospheric chemistry of tropospheric ozone formation is complex. It is initiated by the photodissociation of NO_2_ by solar radiation to form oxygen atoms, and subsequent reactions of these with hydrocarbons to form ozone in chain reactions [33]. Thus, the observed diurnal variation in the surface ozone is primarily attributed to the photochemical process and the diurnal variation in the solar cycle. A full understanding of these factors needs to be developed for Abuja and other cities in Nigeria. This knowledge is needed so that days of high O_3_ episodes can be better predicted which would enable control measures to be taken and health warnings issued.

## Conclusion

These results reveal that ozone concentrations in Abuja frequently exceeded WHO-recommended air quality limits, indicating that most of the residents leaving in Abuja are exposed to an unsafe pollution level from ozone, which is likely to be particularly harmful to sensitive and vulnerable groups.

Based on this result, we recommend that NIMET, the (regulatory and enforcement agency needs to re-evaluate current regulations on air quality monitoring and develop more robust monitoring mechanisms. This will help understand the most effective and efficient measures to improve air quality. There is also a need for a national drive for renewable energy to help reduce the use of private generators. Such actions could be the basis for cleaner air initiatives and ensuring a less polluting environment for city residents, workers and visitors. Further work to determine the types of VOC components which predominate in Abuja is also urgently required.

## Data Availability

**(ADM)**: Data is available on request as agreed with NIMET.
